# The impact of tenofovir disoproxil fumarate on kidney function: four-year data from the HIV-infected outpatient cohort

**DOI:** 10.7448/IAS.17.4.19565

**Published:** 2014-11-02

**Authors:** Nadine Monteiro, Margarida Branco, Susana Peres, Fernando Borges, Kamal Mansinho

**Affiliations:** Infectious Diseases and Tropical Medicine, Hospital Egas Moniz, Lisbon, Portugal

## Abstract

**Introduction:**

With improvements in survival and disease progression in the era of combined antiretroviral therapy, complications such as kidney disease are becoming increasingly prevalent in HIV-infected patients. Tenofovir disoproxil fumarate (TDF) has been associated with nephrotoxicity, including decline in glomerular filtration rate, proximal tubular damage and acute kidney injury.

**Objective:**

Characterize kidney safety of TDF-containing antiretroviral treatment (ART) regimens in HIV-infected patients.

**Methods:**

Non-controlled, observational, retrospective study was based on the clinical files registry of HIV patients who started TDF between January and December 2008. We assessed outpatients followed at a single Portuguese center. Demographic, clinical, virological and immunological data at baseline were collected. Serum creatinine, estimated glomerular filtration rate (eGFR) and creatinine clearance (CrCL) were assessed at baseline, after six months and every year up to four years. CrCL and eGFR were calculated by Cockroft–Gault and Modification of Diet in Renal Disease equations, respectively.

**Results:**

A total of 176 patients (71.6% males) with a mean age of 43 years were enrolled. Ninety-six (52%) were ART-naive patients at TDF initiation. At baseline 12.5% had hypertension, 4% diabetes, 25% chronic hepatitis C and 9% chronic hepatitis B infections; 58% had normal renal function (eGFR ≥90 ml/min/1.73 m^2^), 36% had mild (eGFR 60-89 ml/min/1.73 m^2^) renal dysfunction and 2.3% had moderate (eGFR 30-59 ml/min/1.73 m^2^) renal dysfunction at initiation of TDF. Eighty-three (47%) patients were on protease inhibitors and the remaining on NNRTIs containing regimens. During 48 months follow-up, 5% experienced moderate renal dysfunction and 1.7% severe renal dysfunction. Twenty-one (12%) patients met the definition criteria of rapid decline of renal function (annual decline of eGFR ≥3 ml/min/1.73 m^2^ in two consecutive years). The development of kidney events was associated with age above 50 years, presence of comorbidities and advanced stage HIV infection (p>0.05 in univariate analysis).

**Conclusions:**

These data reveal a favourable renal safety profile of TDF, during a four-year follow-up. Screening for kidney disease markers, regular follow-up and control and prevention of risk factors for renal failure are crucial for adequate management of HIV-infected patients.

